# Dr. Gustavus Pierce English

**Published:** 1917-04

**Authors:** 


**Dr. Gustavus Pierce English,** L. I. College Hospital 1882, died at his home in Utica, Feb. 18, aged 59. He was a laryngologist.

